# Salivary Anionic Changes after Radiotherapy for Nasopharyngeal Carcinoma: A 1-Year Prospective Study

**DOI:** 10.1371/journal.pone.0152817

**Published:** 2016-03-31

**Authors:** Edmond H. N. Pow, Zhuofan Chen, Dora L. W. Kwong, Otto L. T. Lam

**Affiliations:** 1 Oral Rehabilitation, Faculty of Dentistry, The University of Hong Kong, Hong Kong, China; 2 Department of Oral Implantology, Guanghua School of Stomatology, Hospital of Stomatology, Sun Yat-sen University, Guangzhou, China; 3 Department of Clinical Oncology, Li Ka Shing Faculty of Medicine, The University of Hong Kong, Hong Kong, China; School of Medicine and Health Sciences, University of North Dakota, UNITED STATES

## Abstract

**Objectives:**

To investigate the salivary anionic changes of patients with nasopharyngeal carcinoma (NPC) treated by radiotherapy.

**Material and Methods:**

Thirty-eight patients with T1-4, N0-2, M0 NPC received conventional radiotherapy. Stimulated whole saliva was collected at baseline and 2, 6 and 12 months after radiotherapy. Salivary anions levels were measured using ion chromatography.

**Results:**

A reduction in stimulated saliva flow and salivary pH was accompanied by sustained changes in anionic composition. At 2 months following radiotherapy, there was a significant increase in chloride, sulphate, lactate and formate levels while significant reductions in nitrate and thiocyanate levels were found. No further changes in these anion levels were observed at 6 and 12 months. No significant changes were found in phosphate, acetate, or propionate levels throughout the study period.

**Conclusions:**

Conventional radiotherapy has a significant and prolonged impact on certain anionic species, likely contributing to increased cariogenic properties and reduced antimicrobial capacities of saliva in NPC patients post-radiotherapy.

## Introduction

Nasopharyngeal carcinoma (NPC) is a squamous cell carcinoma, with a predilection for certain ethnic groups, such as the southern Chinese population, as well as the Inuit population in Alaska [1]. NPC is especially common in Hong Kong, where the age-adjusted incidence is 14.9 per 100,000 for males and 4.8 per 100,000 for females [2]. Radiotherapy remains the mainstay of treatment for NPC. As the oral cavity and associated stomatomasticatory structures are encompassed within the irradiation field, a wide variety of adverse effects on oral health have been reported, including ulcerative lesions and secondary infections, osteoradionecrosis, fibrosis of the jaw muscles, and salivary hypofunction [3–5].

Salivary hypofunction has a number of adverse impacts on oral and general health, and has been associated with an increased risk of dental caries and discomfort when wearing dentures, as well as difficulties with chewing, swallowing, and speaking. Profound impacts on life quality, as a result of irreversible salivary gland damage, have also been documented following radiotherapy for NPC patients [3–5]. Quantitative reductions in salivary gland function following radiotherapy have been well demonstrated, with dramatic reductions in salivary flow [6, 7]. There is a lack of data, however, documenting qualitative changes in saliva after radiotherapy. A general increase in acidity due to decreased buffering capacity, as well as increased salinity [6, 8], and decreased protein and amylase concentrations [9, 10] have been reported. To our knowledge, no studies have reported salivary anionic changes in NPC patients after radiotherapy [11]. This is pertinent as certain anionic species have major influences on the antimicrobial capacities and cariogenic properties of saliva. For instance, organic anions such as lactate, acetate, and propionate cause rapid decreases in pH and the demineralization of dental hard tissues [11, 12], while phosphate anions contribute to the buffering capacity of saliva and tooth remineralization [13]. Moreover, phosphate anions have an important role in the antimicrobial capacity of saliva, as they have been demonstrated to inhibit the activities of salivary proteins such as apo-lactoferrin [14, 15]. Nitrate has been shown to be an important source of salivary nitrite and nitric oxide, which provide protection against microbial pathogens in the oral cavity and digestive tract [16]. Likewise, the salivary peroxidase system (SPS), of which thiocyanate is an integral component, also contributes to innate defenses. Additionally, the SPS serves to inactivate a number of carcinogenic compounds, and prevents the toxic build-up of hydrogen peroxide [17]. Thus, salivary anions play a critical role in the maintenance of oral health, and an elucidation of their status in NPC patients may provide a greater insight into the qualitative changes in saliva following radiotherapy, and inform further advances in oral health care for this vulnerable patient group.

### Objective

To investigate salivary anionic changes in patients with nasopharyngeal carcinoma (NPC) treated by radiotherapy.

## Materials and Methods

A prospective study design was employed. Consecutive, newly diagnosed NPC patients requiring conventional radiotherapy were recruited from the Department of Clinical Oncology, Queen Mary Hospital, Hong Kong. Patients who had a history of chemotherapy or radiotherapy in the head and neck region were excluded. Written informed consent was provided by all subjects prior to participation in the study, and ethics approval was obtained from the Institutional Review Board of the Li Ka Shing Faculty of Medicine, University of Hong Kong. Stimulated whole saliva (SWS) samples were collected by asking patients to chew on a sterile rubber ring (2mm x 8mm diameter) for 5 minutes and expectorating the saliva into a sterile vial [18]. A pH meter (Sentron 501 Pocket FET) was used to measure salivary pH immediately after collection. Patients were evaluated at baseline (prior to radiotherapy), and at 2, 6, and 12 months post-radiotherapy.

Saliva samples were centrifuged for 10 minutes at 930 x g at 23°C as previously described [19], and analyzed by ion chromomatography (DX-100, Dionex, Sunnyvale, CA, USA) [11]. In brief, the eluents utilized comprised 40 mmol/l NaOH (Eluent 1) and 1.0 mmol/l NaOH (Eluent 2), and samples were run using an anion guard column (IonPac AG11-HC), self-regenerating suppressor (ASRS-Ultra-II), anion separator column, and conductivity detector. Calibration and linearity checks were performed with standard solutions according to a previously established protocol [11, 19], and chromatographic signals were calculated using Peaknet 6.2.

A complete case analysis was performed. Friedman tests were used to compare overall differences in median saliva anion concentration, pH, and flow rate over the four time points. Post-hoc analyses were performed by Wilcoxon signed rank tests. Additional univariate analyses of possible explanatory factors (age, gender, tumor stage, radiation dose) for changes in anion concentrations at two months following radiotherapy were performed via linear regression. Variables with a P value ≤ 0.100 were entered into a forward Wald multiple linear regression model for determination of significant factors. The statistical level chosen was 0.05, and all tests were performed using PASW 18.0 for Windows.

## Results

Forty-two consecutive newly diagnosed Chinese NPC patients were recruited. Four dropouts were documented, with two patients unavailable for review due to scheduling conflicts. One patient had persistent disease following treatment, and another patient experienced disease recurrence. A total of 38 patients completed all four review assessments, and were included in the analyses. Patient age ranged from 32 to 75 (mean age = 53), and all patients had been diagnosed with undifferentiated or poorly differentiated carcinoma. Additional patient and treatment characteristics are presented in Table 1.

**Table 1:** Patient characteristics and radiotherapy parameters.

Characteristics and treatment parameters (n = 38)	No. of patients (%)
Age (years)	
Mean (SD), Range	53 (11), 32 to 75
Gender	
Male	29 (76.3)
Female	9 (23.7)
AJCC tumour stage	
I	5 (13.2)
II	19 (50.0)
III	12 (31.6)
IV	2 (5.2)
T classification	
1	9 (23.7)
2	23 (60.5)
3	5 (13.2)
4	1 (2.6)
N classification	
0	16 (42.1)
1	15 (39.5)
2	7 (18.4)
Radiation dose (Gy)	
6800	18 (47.4)
7800	20 (52.6)

AJCC: American Joint Committee on Cancer

T: Primary tumor

N: Regional lymph nodes

SWS flow rates were significantly reduced at 2 months (median SWS = 0.060 ml/min) following radiotherapy, compared to baseline (median SWS = 0.846 ml/min). No significant changes in SWS flow rates were observed between the 2 month evaluation and assessments at 6 and 12 months. Salivary pH followed a similar trend, with a significant decrease at 2 months (median pH = 6.166) compared to baseline (median pH = 6.953), and a subsequent plateau thereafter. At 2 months after radiotherapy, there were significant increases in chloride (p<0.001), sulphate (p<0.001), lactate (p<0.001) and formate levels (p = 0.011), with concomitant reductions in nitrate (p = 0.015) and thiocyanate (p<0.001) levels (Table 2).

**Table 2:** SWS anionic concentrations (mmol/L) at baseline and 2, 6, and 12 months after radiotherapy (n = 38).

	BL	2 months	6 months	12 months	Time*	Time^
					p-value	Multiple Comparisons
Chloride	27.31(16.81–42.78)	101.37(83.75–110.73)	94.77(74.05–113.59)	96.20(82.71–109.64)	<0.001	(BL) < (2), (6), (12)
Nitrate	0.21(0.07–0.75)	0.07(0.04–0.20)	0.06(0.04–0.14)	0.07(0.04–0.13)	0.015	(BL) > (2), (6), (12)
Phosphate	3.58(2.25–4.53)	3.74(3.11–5.26)	4.15(2.87–4.98)	3.77(3.38–6.13)	n.s.	-
Sulphate	0.10(0.08–0.16)	0.25(0.20–0.34)	0.22(0.16–0.31)	0.24(0.16–0.31)	<0.001	(BL) < (2), (6), (12)
Thiocyanate	0.30(0.17–0.44)	0.08(0.00–0.20)	0.06(0.00–0.19)	0.08(0.00–0.27)	<0.001	(BL) > (2), (6), (12)
Lactate	0.01(0.00–0.08)	0.31(0.03–0.56)	0.15(0.04–0.61)	0.13(0.03–0.66)	<0.001	(BL) < (2), (6), (12)
Acetate	0.54(0.36–0.98)	0.68(0.41–0.92)	0.59(0.18–1.23)	0.51(0.22–0.87)	n.s.	-
Propionate	0.05(0.02–0.11)	0.07(0.02–0.15)	0.06(0.02–0.18)	0.07(0.02–0.12)	n.s.	-
Formate	0.01(0.01–0.05)	0.03(0.01–0.09)	0.04(0.01–0.07)	0.04(0.02–0.10)	0.011	(BL) < (2), (6), (12)

BL = baseline,

*Friedman test comparing the differences of medians over the four time points

^Wilcoxon signed rank test comparing the differences of medians between two time points

Among these anions, increased lactate concentrations at 2 months were significantly associated with a more advanced tumor stage (adjusted R^2^ = 0.110, B = 0.221, p = 0.024), while no associations were found with age, gender, or radiation dose. No significant changes in phosphate, acetate, or propionate levels were observed over the course of the study period.

## Discussion

Saliva plays a vital role in both oral and general health. It contains a plethora of antimicrobial components which act as an important line of defense against oral pathogens and infection, as well as a buffering system which contributes to the remineralization of tooth enamel. It also acts as a lubricant and protects the oral mucous membranes, and is involved in taste, mastication, swallowing, and digestion [20]. SWS flow was significantly reduced in NPC patients following radiotherapy, with an accompanying increase in saliva acidity. These effects were sustained following radiotherapy, and persisted for at least 12 months. Such impacts on salivary function have been widely demonstrated in this patient group, and have been shown to have adverse consequences for oral health and patient quality of life [3, 4, 6, 8].

Saliva is formed in a two stage process prior to secretion into the oral cavity. The primary fluid has a tonicity comparable to that of plasma, and is transported across cell membranes and junctions in the acini. Subsequently, this primary fluid is modified and becomes hypotonic with the active and partial reabsorption of sodium, chloride, and bicarbonate by the ductal epithelium [10]. These salivary ions are subject to Heidenhain’s law, which stipulates that their concentrations increase and subsequently plateau with increased salivary flow rates. At high flow rates due to stimulation, the primary fluid passes through the ductal systems too quickly for the same degree of reabsorption, resulting in a saliva with increased salt concentration [6]. Conversely, concentrations of these ions are decreased with lowered salivary flow rates. Radiation damage, however, severely compromises the reabsorptive abilities of salivary gland ductal epithelium [21], and significantly increased sodium [6, 8, 9, 22], chloride [21], and bicarbonate concentrations have been reported in saliva collected from patients undergoing radiotherapy. Correspondingly, an increase in chloride concentration was documented for up to 12 months in the present study. Higher salivary salt concentrations may have implications for the dental hard tissues, as NaCl has been shown to have a promoting effect on demineralization of enamel and dentine by organic acids such as lactate, formate, propionate, and acetate [23].

Increases in salivary organic acid concentrations have not been previously reported following radiotherapy. While lactate has been detected in saliva collected directly from the parotid glands [24], the oral flora is considered to be the major source of organic acids, which are metabolites produced by the bacterial breakdown of carbohydrates [25]. A greater oral microbial load associated with increased dental plaque following radiation therapy may have been a possible cause of increased salivary lactate levels in the present study. It must be noted, however, that decreased clearance of oral bacteria [26] and their organic acid metabolites [25], due to a concomitant reduction in salivary flow, may also have been a contributory factor. The significant increase in lactate concentration observed in the present study is notable, given that this acid has been shown to be associated with higher rates of demineralization than other organic acids such as acetate and propionate [12]. This is especially pertinent given the association of increased salivary lactate levels with a more advanced tumor stage, and highlights the importance of oral care in this patient group following radiotherapy.

Salivary sulfate concentrations detected at baseline (0.10 mmol/L) closely matched values (0.072 mmol/L, range 0.052–0.107mmol/L) previously reported in stimulated saliva samples collected from healthy adults [27]. As Cole and Landry [27] found sulfate concentrations in saliva samples to be substantially lower than in serum samples, it was speculated that sulfate, similarly to sodium, is selectively resorbed by ductal epithelium in the salivary glands. This is supported by the observation of significantly increased sulfate concentrations with irradiation damage to the salivary glands in the present study, which appears to be the first to report salivary sulfate concentrations following irradiation.

The role of dietary nitrates has been increasingly recognized in affording protection against cardiovascular diseases [28]. Dietary nitrates also have a role in host defense against pathogens [16]. Together with the kidneys, the salivary glands play a major role in systemic nitrate regulation. The salivary glands are able to concentrate up to a quarter of the circulating nitrate [29], and actively secrete bloodstream nitrate into saliva [30]. The significant decrease in salivary nitrate in the present study is supported by a prior short-term (six week) study [31] conducted amongst nasopharyngeal carcinoma patients undergoing intensity modulated radiotherapy, which attributed reduced salivary nitrate levels to irradiation-induced effects on active secretion of nitrate by the salivary glands, rather than reductions in salivary volume per se. Given the importance of nitrates to general health, the finding of a sustained reduction extending to at least 12 months following radiotherapy is of concern, and appropriate management protocols are worthy of further investigation.

A reduction of salivary thiocyanate levels was also observed. While Edgar *et al*. [32] have previously documented decreases in thiocyanate levels in irradiated monkeys, the present study appears to be the first to report the reduction of this anion in humans following radiotherapy. Thiocyanate, along with peroxidase and hydrogen peroxide, form the salivary peroxidase system, which has an integral role in innate immune defense against microbes in the oral cavity [33]. The method of saliva collection may have affected the results observed in the present study, as increased saliva flow produced by stimulation has been shown to decrease salivary thiocyanate [34]. Nevertheless, the thiocyanate concentration documented at baseline (0.30 mmol/L) corresponded well with values (0.34±0.20 mmol/L) obtained by a prior study utilizing SWS samples collected from adolescents [34].

The present study appears to be the first to report salivary anionic changes following radiotherapy in NPC patients, and utilized a protocol for ion chromatography previously demonstrated to be valid and reliable in healthy subject groups [11, 19, 35]. The prospective design of this study permitted the determination of anion levels at regular and scheduled time points over the 12 month study period. The study cohort was homogeneous with respect to mode of therapy, enabling the assessment of impacts in NPC patients treated with conventional radiotherapy. The data obtained could serve as a baseline for comparisons with alternative management protocols such as intensity modulated radiotherapy and treatment incorporating the use of radio-protectants [5, 36].

### Study limitations

Flow rate has an important influence on variation in electrolyte composition and concentration in saliva [37], and studies which assess stimulated saliva have reported greater inter- and intra-subject variance compared to unstimulated saliva [38]. Unstimulated saliva may also have a greater relevance for dental health, as it is constantly in contact with oral tissues [11]. Collection and analysis of unstimulated saliva, however, would not have been practical in the present study, due to the deleterious effects on salivary gland function following radiotherapy. Indeed, it has previously been reported that unstimulated salivary flow rate may be less than 10ul/min following radiotherapy [21]. An additional limitation with the use of whole saliva, either stimulated or unstimulated, was the likely contribution of constituents within the oral cavity to the salivary anionic composition. Constituents of whole saliva are contributed not only by the salivary glands, but also gingival crevicular fluid, bacteria, and inflamed mucosal tissues after radiotherapy [22, 39]. It is widely recognized that plaque fluid comprises substantial levels of organic acidic anions produced through bacterial metabolism [40], and it has previously been suggested that thorough scaling and prophylaxis prior to saliva collection may improve standardization and remove the contribution of organic acids to saliva from dental plaque [11]. While scaling and an accounting of potentially confounding oral health factors was not feasible in the present study, it is notable that organic acid concentrations were higher at baseline, compared to levels previously documented in a healthy subject group [11] which did receive a dental cleaning by a registered dentist prior to saliva collection.

While previous studies have documented the dramatic reduction of salivary flow and buffering capacity of saliva post-radiotherapy [3, 6, 8], the results of this study suggest that changes in certain salivary anionic species following radiotherapy in NPC patients are prolonged, and that this may further contribute to the lowered protective effects of saliva. Clinical management of xerostomia remains a challenge in NPC patients following radiotherapy [41, 42], with a recent evidence-based review suggesting that no current topical therapies are effective for relieving symptoms associated with dry mouth [43]. The present study provides further insight into the changes in salivary chemistry following irradiation, with a view to contributing to the development of artificial saliva for use in the management of radiation-induced hyposalivation and xerostomia.

## Conclusion

Conventional radiotherapy was observed to have a significant impact on the anionic composition of stimulated whole saliva in NPC patients. The documented changes in anionic species were persistent for at least one year, and may further contribute to the increased cariogenic properties and reduced antimicrobial capacities of saliva post-radiotherapy.

## Supporting Information

S1 Table Subject Data. (PDF)
