# Supplementary material for: Salivary Anionic Changes after Radiotherapy for Nasopharyngeal Carcinoma: A 1-Year Prospective Study
Source: PLoS One. 2016 Mar 31;11(3):e0152817. doi: 10.1371/journal.pone.0152817 (PMC4816308; doi:10.1371/journal.pone.0152817)
Supplement: S1 Table — (PDF) [file pone.0152817.s001.pdf]

S1 Table. Subject Data.

Anion Concentrations (mmol/L)

Anion	Subject														
	1	2	3	4	5	6	7	8	9	10	11	12	13	14	15
Chloride 0	14.53	34.51	98.9	13.59	13.93	127.6	28.5	114.21	13.11	9.5	25.57	53.99	21.86	101.82	10.25
Chloride 2	92.61	110.36	107.39	107.28	121.88	115.41	122.29	20.46	28.53	111.85	79.38	86.28	105.71	124.39	93.24
Chloride 6	55.8	69.08	6.68	116.5	90.32	53.27	119.77	22.93	94.02	107.7	91.71	103.6	120.26	73.91	103.17
Chloride 12	105.69	84.13	95.22	103.69	113.28	97.1	101.9	100.11	96.53	92.39	97.61	82.78	91.71	124.81	95.88
Nitrate 0	1.44	0.6	0.18	0.01	0.16	0.14	0.19	0.03	2.06	0.18	0.39	0.43	0.01	0.22	0.01
Nitrate 2	0.82	0.17	0.09	0.12	0.03	0.36	0.02	0.2	0.2	2.66	0.01	5.46	0.04	0.04	0.02
Nitrate 6	0.11	0.06	0.18	0.05	0.02	0.09	0.05	0	0.03	0.1	0.11	0.09	0.03	0.08	0.02
Nitrate 12	0.05	0.15	0.17	0.03	0.04	0.05	1.31	0.04	0.06	0.67	0.04	0.07	0.11	0.05	0.03
Phosphate 0	1.48	5.99	4.08	1.72	1.28	5.29	2.98	2.84	2.96	1.78	3.65	3.48	4.1	3.41	1.73
Phosphate 2	7.79	8.06	5.8	3.59	7.79	5.23	5.1	0.7	1.41	3.14	3.18	4.31	7.28	3.53	3.04
Phosphate 6	3.05	4.64	1.66	4.12	6.58	4.76	5.04	3.88	3.59	4.96	4.43	5.93	3.6	7.85	8.05
Phosphate 12	8.31	6.57	3.7	3.85	8.33	3.42	3.54	2.96	3.74	5.65	3.79	6.47	4.43	3.37	2.79
Sulphate 0	0.02	0.18	0.15	0.05	0.05	0.31	0.12	0.22	0.1	0.07	0.09	0.11	0.08	0.13	0.06
Sulphate 2	0.25	0.3	0.62	0.25	0.11	0.25	0.2	0.06	0.72	0.27	0.17	0.41	0.2	0.2	0.25
Phosphate 6	0.17	0.58	0.05	0.14	0.09	0.11	0.19	0.17	0.25	0.78	0.14	0.23	0.1	0.17	0.13
Phosphate 12	0.19	0.23	0.25	0.15	0.68	0.26	0.16	0.31	0.32	0.87	0.13	0.22	0.14	0.1	0.26
Thiocyanate 0	0.27	0.17	0.14	0.29	0.15	0	0.44	0.6	0.22	0.21	0.39	0.2	0.32	0	0.06
Thiocyanate 2	0.32	0	0.2	0.15	0.04	0	0.1	0.08	0.14	1.12	0.04	0.66	0.02	0	0
Thiocyanate 6	0.04	0	0.14	0.33	0.04	0.16	0.09	0.35	0.05	0.3	0.12	0.11	0.06	0.78	0
Thiocyanate 12	0.27	0	0.13	0.41	0	0	0.37	0.5	0.1	0.3	0.11	0.05	0.12	0	0
Lactate 0	0.01	0	0.09	0	0.01	0.36	0.01	0.15	0.31	0.02	0.01	0	0.01	0.01	0
Lactate 2	0.02	0.39	0.47	0.3	0.08	1.07	0.8	0.17	0.66	0.59	0.15	0.36	0.31	0.3	0.14
Lactate 6	0.05	0.48	0.01	1.38	0.08	0	0.17	0.11	0.12	2.87	0.15	0.38	0.09	0.02	0
Lactate 12	0.03	0.36	1.08	2.08	2.66	1.07	0.14	0.03	0.78	1.63	0.11	0.09	0.15	0.02	0.03
Acetate 0	0.04	1.65	0.25	0.43	0.12	0.33	0.97	0.41	1.15	0.29	0.33	0.47	0.53	0.46	0.69
Acetate 2	2.03	0.14	0.53	0.7	0.2	0.24	0.63	0.32	2.75	0.63	0.7	0.82	0.44	0.11	1.22
Acetate 6	0.7	0.12	0.47	0.27	0.45	1.61	0.19	2.62	0.77	0.84	0.59	1.23	0.24	2.41	0.09
Acetate 12	0.54	0.18	0.77	0.35	0.85	0.36	0.19	0.79	0.82	0.16	1.66	0.33	0.59	0.02	0.43
Propionate 0	0	0.24	0.02	0.04	0.01	0.05	0.05	0.24	0.01	0.04	0.02	0.01	0.03	0.05	0.03
Propionate 2	0.08	0	0.02	0.05	0.01	0.01	0.06	0.08	0.2	0.09	0.07	0.09	0.02	0	0.3

Propionate 6	0.04	0	0.1	0.05	0.06	0.27	0.03	0.59	0.09	0.1	0.08	0.17	0.04	0.31	0.01
Propionate 12	0.09	0.01	0.05	0.07	0.07	0.06	0.04	0.24	0.07	0	0.18	0.07	0.08	0	0.01
Anion	Subject														
	16	17	18	19	20	21	22	23	24	25	26	27	28	29	30
Formate 0	0.01	0.01	0.08	0.01	0.01	0.13	0.01	0.19	0.14	0.01	0.01	0.02	0.01	0.06	0.01
Formate 2	0.03	0.04	0.06	0.01	0.03	0.07	0.12	0.09	0.37	0.11	0.11	0.28	0.04	0.03	0.02
Formate 6	0.07	0.07	0.04	0.02	0.06	0.05	0.04	0.01	0.27	0.15	0.05	0.08	0.03	0.02	0
Formate 12	0.08	0.05	0.02	0.08	0.06	0.1	0.1	0.24	0.2	0.05	0.09	0.23	0.05	0.02	0.01
Chloride 0	24.09	14.7	26.12	17.18	39.15	7.78	22.95	32.1	31.29	17.69	41.95	30.21	23.96	29.24	15.71
Chloride 2	100.22	92.88	108.58	105.77	82.71	79.98	101.32	106.02	19.19	18.41	104.67	106.49	116.95	87.92	127.2
Chloride 6	95.53	84.64	56.11	110.52	90.6	14.66	113.45	133.1	134.49	122.45	134.14	104.38	122.58	74.1	107.98
Chloride 12	60.04	37.88	92.61	107.99	4.89	128.01	133.06	89.3	76.74	145.9	98.06	87.09	108.42	118.07	82.5
Nitrate 0	0	0.37	0.84	0.01	2.36	0.01	0.15	5.48	1.48	0.37	0.22	0.51	0.18	0.72	0.28
Nitrate 2	0.07	0.07	0.07	0.11	0	0.16	0.07	0.04	0.63	0.29	0.03	0.5	0.05	0.04	0.21
Nitrate 6	0.06	1.24	0.1	0.63	0.06	0.15	0.37	0.04	0.14	0.06	0.04	0.07	0.03	0.06	0.04
Nitrate 12	0.04	1.1	0.09	0.58	0.02	0.03	0.09	0.03	0.72	0.05	0.11	0.07	0.17	0.09	0.04
Phosphate 0	3.34	2.05	4.1	3.54	3.26	1.5	3.71	5.4	4.94	4.97	3.04	4.87	3.89	2.31	3.62
Phosphate 2	6.72	2.88	7.81	4.68	3.73	3.76	8.03	3.34	0.96	0.78	4.01	3.58	2.91	3.64	4.9
Phosphate 6	5.91	2.44	2.65	3.24	4.26	0.81	8.11	7.34	4.2	4.55	4.71	7.86	4.19	3.26	4.27
Phosphate 12	3.14	1.3	5.35	7.99	1.12	8.51	10.08	3.74	3.38	6.01	3.73	4.69	7.79	4.62	3.22
Sulphate 0	0.15	0.08	0.08	0.09	0.13	0.07	0.12	0.12	0.1	0.09	0.1	0.13	0.07	0.09	0.08
Sulphate 2	0.35	0.36	0.37	0.26	0.21	0.26	0.34	0.3	0.22	0.24	0.06	0.34	0.19	0.17	1.08
Phosphate 6	0.26	0.33	0.67	0.26	0.2	0.29	0.53	0.27	0.21	0.2	0.14	0.22	0.21	0.59	0.41
Phosphate 12	0.67	0.28	0.34	0.2	0.13	0.09	0.34	0.23	0.31	0.13	0.22	0.24	0.26	0.17	0.76
Thiocyanate 0	0.84	0.39	0.32	0.04	0.88	0.19	0.27	0.21	0.44	1.23	1.25	0.45	0.5	0.41	0.19
Thiocyanate 2	0.34	0	0.2	0	0	0.11	0	0	0.17	0.63	0.03	0.2	0.1	0	0.24
Thiocyanate 6	0.04	0.22	0	0.08	0.03	0.18	0.04	0	0.12	0.22	0	0	0.22	0	0
Thiocyanate 12	0.29	0.74	0.18	0.13	0.04	0.04	0.09	0	0.43	0.11	0	0.04	0.62	0	0
Lactate 0	0	0	0	0.01	0.08	0.01	0.01	0	0	0.25	0.01	0.34	0.03	0.02	0
Lactate 2	0.07	1.05	0.03	0.2	0.31	2.17	1.51	0	0.34	0.01	0.02	0.47	0	0	0.55
Lactate 6	0.11	0	0	0.15	0.08	0.62	0.72	0	0.69	0.22	0.04	0.6	0.16	0	0.39
Lactate 12	0	0	0.04	0.11	0.38	1.01	0.38	0	0.62	0.13	0.01	1.53	0.2	0.11	0.14
Acetate 0	1.27	0.56	0.13	0.81	1.25	1.25	0.7	0.42	1.03	0.39	0.48	0.76	0.36	0.22	0.33
Acetate 2	0.91	0.6	0.67	0.78	1.17	0.71	1.29	0.64	0.49	0.87	0.06	0.16	0.93	0.34	0.96
Acetate 6	1.23	1.67	0.13	0.41	0.31	0.61	1.47	0.05	1.19	0.4	0.14	0.16	1.51	0	1.12
Acetate 12	0.38	1.9	0.76	0.61	0.88	0.27	0.86	1.53	2.04	0.43	0.03	0.16	0.92	0.23	0.48
Propionate 0	0.24	0.05	0.01	0.12	0.25	0.22	0.07	0.01	0.1	0.05	0.06	0.06	0.02	0.04	0.02

Propionate 2	0.06	0.14	0.07	0.06	0.19	0.21	0.46	0.02	0.09	0.05	0.01	0.02	0.06	0.05	0.14
Propionate 6	0.09	0.07	0	0.01	0.02	0.05	0.21	0	0.12	0.06	0.01	0.02	0.18	0	0.18
Anion	Subject														
Propionate 12	0.05	0.05	0.07	0.02	0.11	0.04	0.14	0.01	0.54	0.07	0.01	0.02	0.19	0.03	0.1
Formate 0	0.01	0.01	0.02	0.09	0.02	0.01	0.01	0	0.01	0.01	0.02	0.01	0	0	0.01
Formate 2	0.11	0.02	0	0.02	0.09	0.08	0.04	0	0	0.01	0	0.04	0.02	0	0.19
Formate 6	0.12	0.01	0	0	0.04	0.06	0.19	0	0.04	0.02	0	0.07	0.12	0	0.03
Formate 12	0.09	0.01	0	0.02	0.15	0.05	0.23	0	0.04	0.02	0	0.04	0.04	0.01	0.13

Anion	Subject							
	31	32	33	34	35	36	37	38
Chloride 0	94.45	90.17	45.28	33.9	38.96	24.81	85.07	21.74
Chloride 2	25.76	84.09	101.42	89.01	119.77	99.47	21.27	135.18
Chloride 6	98.24	20.83	77.41	79.68	88.77	114.03	82.45	109.61
Chloride 12	76.54	72.78	87.13	5.04	120.67	120.54	125.65	73.09
Nitrate 0	0.07	0.08	1.55	0.05	3.63	2.19	0.07	0.2
Nitrate 2	0.04	0.25	0.06	0.04	0.02	0.01	0.07	0.13
Nitrate 6	0.84	2.88	0.66	0.03	0.03	0.05	0.07	0.59
Nitrate 12	0.39	0.06	0.13	0.04	0.03	0.12	0.1	0.05
Phosphate 0	3.98	1.95	5.36	5.3	4.61	2.07	4.5	4.38
Phosphate 2	3.28	2.59	4.06	5.33	3.91	3.62	0.47	4.89
Phosphate 6	2.65	1.8	2.86	3.57	2.88	3.6	2.32	1.08
Phosphate 12	5.08	2.72	3.62	0.35	8.75	3.47	4.2	3.49
Sulphate 0	0.28	0.25	0.19	0.19	0.19	0.1	0.18	0.1
Sulphate 2	0.11	0.28	0.23	0.27	0.34	0.14	0.15	0.29
Phosphate 6	0.64	0.08	0.31	0.3	0.23	0.22	0.18	0.55
Phosphate 12	0.23	0.28	0.26	0.13	0.26	0.08	0.19	0.59
Thiocyanate 0	0.37	0	0.35	0.31	0.44	0.32	0.03	0.09
Thiocyanate 2	0.52	0.17	0.09	0.18	0.02	0.02	0	0
Thiocyanate 6	0.66	0.42	0.13	0	0.06	0.03	0.02	0
Thiocyanate 12	0.43	0	0.13	0	0.06	0.03	0	0
Lactate 0	0.32	0.21	0	0	0	0.13	0.02	0
Lactate 2	0	0.32	0.01	0.54	0	0.95	0.19	0.57
Lactate 6	0	0.09	0.23	1.07	3.57	1.57	0.21	0.88

Lactate 12	0	0.94	0	0.01	0.11	0.41	0.31	0
Acetate 0	2.56	1.13	0.65	0.61	0.91	0.48	1.62	0.8
Acetate 2	0.92	0.75	0.53	0.67	2.09	0.04	0.96	0.69
Acetate 6	5.23	0.13	0.73	0.58	1.27	0.46	1.16	0.11
Acetate 12	3.44	0.87	0.09	0.06	1.18	0.08	1.93	0.45
Propionate 0	0.48	0.12	0.02	0.08	0.06	0.02	0.37	0.04
Propionate 2	0.24	0.12	0.05	0.18	0.62	0	0.44	0.12
Propionate 6	0.57	0	0.06	0.12	0.58	0.02	0.45	0.02
Propionate 12	0.41	0.17	0	0.01	0.21	0	0.23	0.12
Formate 0	0.1	0.16	0.01	0.01	0.01	0.05	0.06	0.01
Formate 2	0.01	0.01	0.01	0.02	0.05	0.03	0.19	0.1
Formate 6	0.05	0	0.01	0.11	0.06	0.04	0.25	0.05
Formate 12	0.12	0.03	0	0.01	0.03	0.03	0.02	0.14
